# Regular Vigorous-Intensity Physical Activity and Walking Are Associated with Divergent but not Convergent Thinking in Japanese Young Adults

**DOI:** 10.3390/brainsci11081046

**Published:** 2021-08-06

**Authors:** Chong Chen, Yasuhiro Mochizuki, Kosuke Hagiwara, Masako Hirotsu, Shin Nakagawa

**Affiliations:** 1 Division of Neuropsychiatry, Department of Neuroscience, Yamaguchi University Graduate School of Medicine, Ube, Yamaguchi 755-8505, Japan; khagi@yamaguchi-u.ac.jp (K.H.); hirotsu@yamaguchi-u.ac.jp (M.H.); snakaga@yamaguchi-u.ac.jp (S.N.); 2 RIKEN Center for Brain Science, Wako, Saitama 351-0198, Japan; mochizuki@brain.riken.jp

**Keywords:** international physical activity questionnaires, exercise, creativity, divergent thinking, alternate uses test, convergent thinking

## Abstract

The beneficial effects of regular physical activity (PA) on cognitive functions have received much attention. Recent research suggests that regular PA may also enhance creative thinking, an indispensable cognitive factor for invention and innovation. However, at what intensity regular PA brings the most benefits to creative thinking remains uninvestigated. Furthermore, whether the levels of regular PA affect the acute PA effects on creative thinking is also unclear. In the present study, using a previous dataset that investigated the effects of an acute bout of aerobic exercise on creative thinking in healthy Japanese young adults (22.98 ± 1.95 years old) in the year 2020, we tested the association between different intensities of regular PA (i.e., vigorous, moderate, and walking) and creative thinking with the cross-sectional baseline data using multiple linear regression. We also investigated whether regular PA levels were associated with the acute aerobic exercise intervention effects on creative thinking. The results showed that cross-sectionally, the regular PAs were differentially associated with divergent but not convergent thinking. Specifically, whereas the amount of vigorous-intensity PA was positively associated with fluency and flexibility, the amount of walking was positively associated with novelty on the alternate uses test (AUT) measuring divergent thinking. Importantly, the explained variances of fluency, flexibility, and novelty were 20.3% (*p* = 0.040), 18.8% (*p* = 0.055), and 20.1% (*p* = 0.043), respectively. None of the regular PAs predicted convergent thinking (i.e., an insight problem-solving task), nor were they associated with the acute aerobic exercise intervention effects on divergent and convergent thinking. These findings suggest that engaging in regular vigorous-intensity PA and walking may be useful strategies to enhance different aspects of divergent thinking in daily life.

## 1. Introduction

The World Health Organization (WHO) recommends that adults aged 18–61 years should conduct at least 150 min of moderate-intensity physical activity (PA), 75 min of vigorous-intensity PA, or an equivalent combination of both moderate- and vigorous-intensity PA [1]. In line with this recommendation, a growing body of research has confirmed that engaging in regular PA enhances a wide range of cognitive functions, including attention, executive functions, memory storage and retrieval, and so on (for reviews and meta-analyses, [2,3,4,5]). Potential neurobiological mechanisms of such benefits include the activation of the prefrontal cortex and the release of lactate, cortisol, neurotrophins, and neurotransmitters (see the above reviews for details). However, little is known about the effects of PA on creative thinking.

Creative thinking is generally believed to comprise two fundamental cognitive processes: divergent and convergent thinking [6,7,8]. Divergent thinking involves generating multiple, novel solutions and is commonly assessed by the alternate uses test (AUT, [9]) or the Torrance Tests of Creative Thinking [10]. In the AUT, for instance, subjects are asked to write down as many as possible unique, original uses of common objects such as “brick” and “umbrella”. The number of generated uses (known as fluency), the number of different conceptually categories of the uses (known as flexibility), and the rareness of the uses (known as originality) are frequently used as indicators of divergent thinking. In contrast, convergent thinking involves approaching a single correct solution [6] and is often assessed by the remote associates test (RAT, [11]), the compound remote associates test (CRA, [12]), or insight problem-solving quizzes (e.g., matchstick arithmetic problems, [13]). In the CRA, subjects are asked to think of a single word that is associated with each of three given words, for instance, “time”, “hair”, and “stretch” (the answer is “long”). In the matchstick arithmetic problem, several sticks form a false arithmetic equation and subjects are asked to move a single stick to make the equation correct. It has been suggested that divergent and convergent thinking differ in such a way that the latter relies more on top-down, executive control [14]. Since both divergent and convergent thinking are indispensable for invention and innovation [6,7,8], the development of effective strategies to enhance creative thinking may have great social significance.

We have recently conducted a systematic review of studies on acute PA and creative thinking and concluded that acute PA has the potential to enhance divergent and convergent thinking (see Introduction and Supplementary Materials of Aga et al. (2021) [15]). For instance, a recent study showed that compared to stay seated, a 4-min walk on a treadmill or outdoors increased the originality of divergent thinking, without affecting performance on CRA that measured convergent thinking [16]. However, acute PA at too low an intensity may not reliably enhance divergent and convergent thinking, while at too high an intensity, it may impair divergent and convergent thinking. We further conducted a randomized controlled trial and showed that a 15-min physical test program improved divergent thinking (in terms of flexibility on the AUT) independent of post-exercise mood [15]. In contrast, the program affected convergent thinking (matchstick arithmetic problems) in a post-exercise mood-dependent way: it tended to enhance convergent thinking in subjects reporting high vigor but impair convergent thinking in those reporting low vigor [15].

However, to the best of our knowledge, the effects of regular or chronic PA on creative thinking has not been systematically reviewed so far. Whereas the effects of acute PA are transient, those of regular PA are long lasting and may be more valuable for long-term enhancement of creative thinking. We, therefore, searched (1) interventional studies that investigated the effects of multiple sessions of PA interventions on creative thinking and (2) observational studies that investigated the association between the levels of regular PA and creative thinking (see the literature search strategy described in the footnote of Table S1). As a result, we identified six interventional studies ([17,18,19,20,21,22], see Table S1) and four observational studies ([23,24,25,26], see Table S2).

To summarize the findings, the identified interventional studies consistently reported significant enhancing effects of PA programs lasting 6–12 weeks (weekly 2–5 sessions) on one or several measures of divergent thinking, such as fluency (i.e., the number of generated uses) on AUT and figural fluency on TTCT [17,18,19,20,21,22]. However, two studies that evaluated convergent thinking with matchstick puzzles failed to find any effects of similar PA programs [17,18]. Similarly, the identified cross-sectional studies consistently found positive associations between self-reported or actigraph-monitored regular PA and measures of divergent thinking in all or a subgroup of subjects [23,24,26] but not convergent thinking (i.e., insight problem-solving quizzes, [25]).

Although the results of our systematic review indicate beneficial effects of regular PA on creative thinking (especially for divergent thinking), the influence of PA intensity or, in other words, at what intensity regular PA brings the most benefits remains unclear. None of the identified interventional studies specified the intensity of their PA programs. Two of the cross-sectional studies did differentiate the intensity of regular PA, but the results were inconclusive. Rominger et al. (2020) [24] reported a significant correlation between total, no-to-light, and moderate but not vigorous and very vigorous everyday bodily movement (EBM) and divergent thinking. However, given the frequently observed correlation among different intensities of regular PA [25], the independent effects of different intensities of regular PA on divergent thinking were unknown in this study. Nakagawa et al. (2020) [25] included measures of walking and moderate- and vigorous-intensity PA in a regression model to predict convergent thinking evaluated with insight problem-solving quizzes but failed to find any significant effects. The lack of evidence on the relation between regular PA intensity and creative thinking is unfortunate because intensity is a critical and frequently discussed issue in PA prescription and planning for enhancing cognitive and mental health [25,27].

Furthermore, none of the studies we reviewed have examined the interaction effect between regular and acute PA on creative thinking. In the field of PA and mood, studies have reported that subjects with high levels of regular PA may show more enhanced mood in response to a new, acute bout of PA [28,29]. However, there were also studies suggesting that this difference only occurs when conducting high- but not low-intensity acute PA [30,31] or that the psychological effects of acute PA are independent of regular PA altogether [32]. Clarifying the interaction effect between regular and acute PA on creative thinking may provide insights into the mechanism by which regular PA enhances creative thinking, and if the interaction effect does exist, it will be worthy to be carefully considered during PA prescription and planning.

Therefore, in the present study, we conducted a secondary analysis of a previously published study [15] that investigated the effects of an acute bout of aerobic exercise on divergent and convergent thinking. Specifically, we tested three hypotheses. First, the cross-sectional associations between regular PA and divergent and convergent thinking are different such that regular PA is only associated with divergent thinking. Second, different intensities of regular PA may have distinct associations with divergent thinking. Third, regular PA may affect the acute aerobic exercise intervention effects on creative thinking. To our knowledge, this is the first study that evaluated the independent effects of different intensities of regular PA on divergent thinking and the first study that investigated whether regular and acute PA interact to affect divergent and convergent thinking.

## 2. Materials and Methods

### 2.1. Participants and Procedure

The study was approved by the Institutional Review Board of Yamaguchi University Hospital and preregistered on the University hospital Medical Information Network Clinical Trial Registry (UMIN-CTR, register ID: UMIN000041122). The study was carried out following the latest version of the Declaration of Helsinki, and all subjects agreed to participate in the study and provided written informed consent. The characteristics of the participants and procedure of the intervention have been described in [15]. In brief, the study was a randomized controlled trial using a between-subjects pre-test–post-test comparison design. Based on a priori power analysis, forty healthy subjects (all undergraduate students, 11 females, 29 males, age: 22.98 ± 1.95 years) were recruited via posters placed on campus and department homepage and through word-of-mouth during the period of July–October 2020. The inclusion criteria were being 20–29 years old at the time of the visit and the exclusion criteria were (1) reporting any history of diseases that greatly affect cardiopulmonary functions, such as chronic heart failure, (2) currently suffering from any mental illness or being scheduled to receive any medical examinations due to suspicion of mental illness, (3) being a member of our department who receives the personnel evaluation directly by the principal investigator of this study, and (4) being judged to be unsuitable for this study (e.g., bodyweight exceeding the applicable weight of the exercise bike). No participant was excluded due to meeting any of the exclusion criteria.

**Figure 1:**
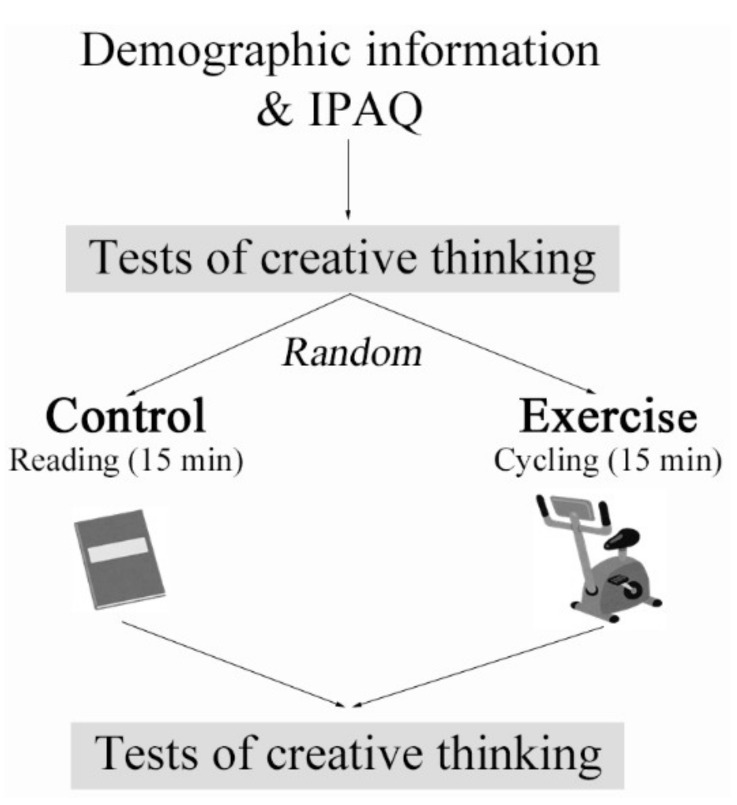
Schematic illustration of the procedure. Adapted from [15].

As shown in Figure 1, subjects first filled out a form assessing their age, sex, education level, regular PA, and so on. Subjects then performed tests of creative thinking at baseline, after which they were randomized to receive either the acute aerobic exercise intervention or control intervention. Immediately after the intervention, subjects rated their mood in terms of pleasure, relaxation, and vigor and, then, conducted tests of creative thinking again.

### 2.2. Measures of Regular PA

The international physical activity questionnaire long-form (IPAQ, [33]) was employed here. The IPAQ has been translated into many languages including Japanese, and its reliability and validity have been established by Craig et al. (2003) [33]. The IPAQ asks subjects to indicate the duration and frequency of different intensities (i.e., walking, moderate, and vigorous) of PA within four different domains (i.e., occupational, transport, household, and leisure related) during the past seven days. To investigate the independent effects of different intensities of regular PA, we calculated the total amount of PA (minutes/week) for each intensity, respectively. Furthermore, we calculated the total weekly PA metabolic equivalents (MET-minutes, hereafter referred to as total PA), by weighting the amount of PA of each intensity (minutes/week) with a MET energy expenditure estimate assigned to each intensity (8 for vigorous, 4 for moderate, and 3.3 for walking).

### 2.3. Measures of Creative Thinking

Divergent thinking was measured with the AUT [9]. In this test, subjects were presented with three common objects and asked to write down as many as possible uncommon, original, and unique uses of those objects within 4 min on an A4 size blank paper. We calculated the fluency, flexibility, and originality for each subject [34,35]. Fluency was the number of generated uses, flexibility was the number of different conceptual categories the uses are from, and originality was the rareness of the generated uses here defined as the number of conceptual categories. After sufficient training [36] under the supervision of the corresponding author, a primary coder scored all responses and a secondary coder scored responses of a randomly selected object; the two coders (both were undergraduate research assistants enrolled in the medical department) reached a substantial or almost perfect agreement, indicated by Cohen’s κ = 0.936 for flexibility and Cohen’s κ = 0.706 for originality.

Convergent thinking was evaluated with the matchstick arithmetic problems developed by Knoblich et al. [13]. At pre-test, six problems were presented, and subjects had 12 min to solve these problems. The unsolved problems were presented again at post-text, together with another set of creative problem-solving puzzles that require visuospatial and logical reasoning [37]. To measure the intervention effects, we created two measures of convergent thinking for data analysis: one was a creative problem-solving (CPS) score consisting of matchstick arithmetic problems at pre-test and creative problem-solving puzzles at post-test; the other was a matchstick re-test score obtained at post-test only and calculated as the proportion of correctly solved problems that they failed at pre-test (subjects that correctly solved all problems at pre-test were removed from this analysis).

### 2.4. Intervention

For the acute aerobic exercise intervention, we used an automated physical test program built in an exercise bike (Wellbike BE-260, Fukuda Denshi, Tokyo, Japan). The program lasted 15 min and consisted of 10 min of physical test and 5 min of cooldown. This was essentially a graded exercise program designed for convenient physical testing. Under this program, subjects sat quietly on the bike during the first minute to measure their resting pulse, after which they started pedaling at a pace of 50 rpm. The workload increased at 4 and 7 min after the start of the program according to the pulse rate of the subjects at the moment. This program was chosen as the exercise intervention because its workload was predicted to fall between normal cycling at moderate intensity and intense cycling with maximal effort, two intensities tested by Colzato et al. (2013) [38]. Exercise at this intensity was believed to improve subjects’ mood and be more likely to enhance creative thinking [15]. Subjects’ heart rate was monitored with an Apple Watch Series 4 (Apple Inc., Cupertino, CA, USA). The mean heart rate was 118.25 (standard deviation or SD 11.93) bpm, the maximal heart rate was 158.65 (SD 13.00) bpm, and the heart rate in the last minute of the program was 102.31 (SD 11.93) bpm. The details of the exercise intervention are available in [15]. For the control intervention, we asked subjects to read mood-neutral materials on the association between PA and brain functions at a self-selected pace. The mean, maximal, and last-minute heart rate under the control intervention were 75.10 (SD 11.27), 89.70 (SD 12.18), and 75.53 (SD 12.40) bpm, respectively. Both interventions were conducted by an undergraduate research assistant under the supervision of the corresponding author.

### 2.5. Statistical Analysis

The statistical analysis was conducted with IBM SPSS Statistics 26.0. To investigate the association between regular PA and creative thinking at baseline, we combined subjects from the exercise and control groups together (*n* = 40). The normality of the data was checked using the Shapiro–Wilk test. Due to non-normal distribution of total PA, Spearman correlation analysis was used to examine the association between total PA and creative thinking at baseline as well as the acute aerobic exercise intervention effects. Confidence intervals (CI) for the Spearman correlation coefficients were calculated based on [39]. Multiple linear regression was used to evaluate the independent effects of different intensities of regular PA on creative thinking at baseline and the acute intervention effects. The normal P-P plot of regression standardized residual was confirmed and shown in Figures S1 and S2. We did not detect any obvious multicollinearity (i.e., variance inflation factors all <5) or homoscedasticity issue with the multiple linear regression. Given that we had a small set of predictors (i.e., three) and that we were not clear which was the best predictor, we used the standard “Enter” method for the multiple linear regression. A significance level of *p* < 0.05 was used.

## 3. Results

### 3.1. Regular PA and Divergent and Convergent Thinking at Baseline

**Figure 2:**
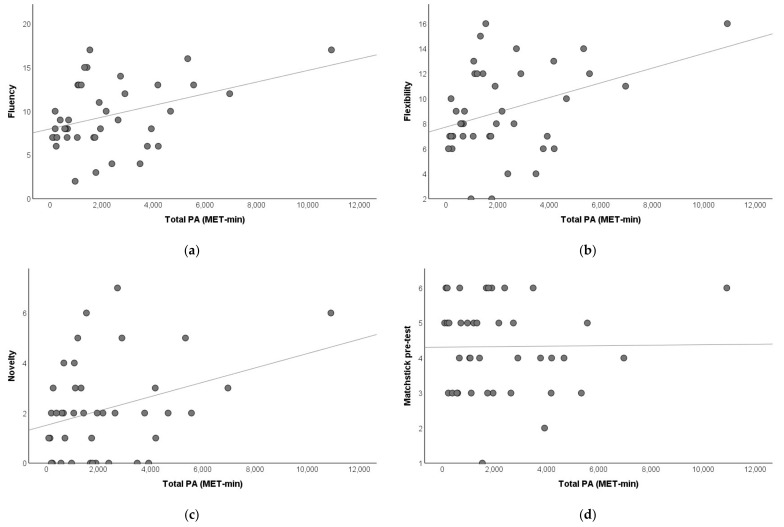
Scatterplot of the association between total PA and creative thinking at baseline. (**a**) Fluency. (**b**) Flexibility. (**c**) Novelty. (**d**) Matchstick pre-test.

Spearman correlation analysis indicated that total PA was marginally associated with fluency (rho = 0.293, 95% CI= [−0.027, 0.559], *p* = 0.067) and flexibility (rho = 0.283, 95% CI = [−0.038, 0.551], *p* = 0.077) but not novelty (rho = 0.237, 95% CI = [−0.085, 0.514], *p* = 0.142) on AUT of divergent thinking (Figure 2). Total PA was not associated with the matchstick pre-test measure of convergent thinking (rho = 0.091, 95% CI= [−0.228, 0.392], *p* = 0.575).

**Figure 3:**
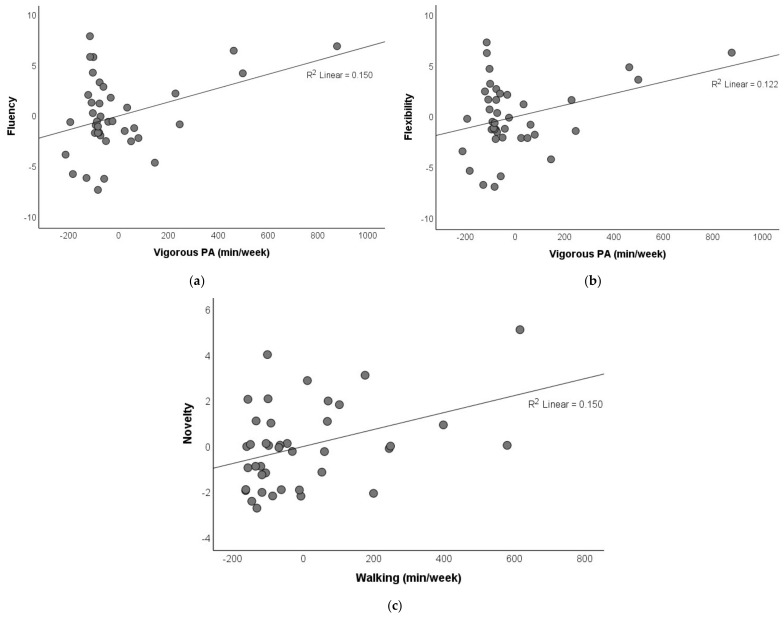
Partial regression plot of the association between different intensities of regular PA and creative thinking at baseline. (**a**) Vigorous-intensity PA and fluency. (**b**) Vigorous-intensity PA and flexibility. (**c**) Walking and novelty.

To investigate the independent effects of different intensities of regular PA on creative thinking at baseline, we incorporated all three intensities of regular PA as independent variables to predict creative thinking using multiple linear regression models (Table 1). The results showed that whereas the amount of vigorous-intensity PA positively predicted fluency (*p* = 0.016) and flexibility (*p* = 0.032), walking positively predicted novelty (*p* = 0.016) on AUT measure of divergent thinking. Notably, the models explained 18.8–20.3% variances of the AUT measures. A partial regression plot of the associations is shown in Figure 3. In contrast, the model incorporating all three intensities of regular PA did not predict convergent thinking (*p* = 0.976).

### 3.2. Regular PA and Acute Aerobic Exercise Intervention Effects

Spearman correlation analysis indicated that total PA was not associated with any of the acute intervention effects on divergent or convergent thinking in either the exercise (all *p* > 0.09) or control (all *p* > 0.50) group.

**Figure 4:**
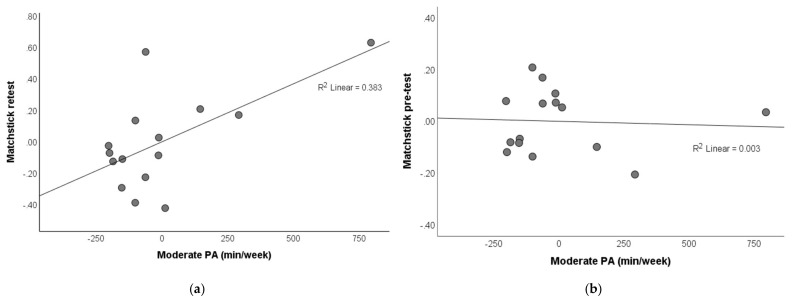
Partial regression plot of the association between moderate-intensity PA and convergent thinking in control group. (**a**) Matchstick retest after the intervention. (**b**) Matchstick pre-test at baseline.

We next included all three intensities of regular PA as independent variables to predict the acute intervention effects using multiple linear regression models (Table 2). The results showed that the amount of different intensities of regular PA did not predict the change in divergent or convergent thinking in response to the acute aerobic exercise intervention (all *p* > 0.30). However, in the control group, the amount of moderate-intensity PA positively predicted the matchstick retest measure of convergent thinking in response to the control intervention (*p* = 0.024, Figure 4a). This was not due to a low score at pre-test, as moderate-intensity PA did not predict matchstick pre-test scores (*p* = 0.856, Figure 4b).

## 4. Discussion

We found that total PA in terms of weekly MET-minutes was marginally correlated with fluency and flexibility but not novelty of divergent thinking nor convergent thinking. These correlations (rho = 0.293 for fluency, rho = 0.283 for flexibility) are considered small in magnitude [40]. More importantly, our multiple linear regression incorporating different intensities of regular PA showed that whereas the amount of vigorous-intensity PA (minutes/week) positively predicted fluency and flexibility, that of walking positively predicted novelty of divergent thinking. In contrast, none of them predicted convergent thinking. These results suggest that the effects of regular PA on divergent thinking are intensity-specific. Thus, in the case of divergent thinking, it is perhaps not the total amount of PA that matters, rather, the intensity of the PA is more important. To enhance divergent thinking, people should conduct more vigorous-intensity PA as well as walking.

Vigorous-intensity PA refers to activities that require hard physical effort and cause large increases in breathing, for instance, heavy lifting, fast bicycling, and intense aerobics. We have previously proposed that higher intensities of PA may bring more benefits to the brain because they generally cause more extensive, enhanced, and long-lasting neurobiological changes [25]. For instance, it has been reported that a single bout of vigorous- but not low-intensity cycling increased the peripheral level of brain-derived neurotrophic factor (BDNF) in young adults [41]. Peripheral BDNF can pass the brain–blood barrier and supports the production, growth, differentiation, and survival of neurons and thereby enhances a large variety of cognitive functions [42]. In rats, higher speed of running has been reported to increase the extracellular concentration of dopamine in the striatum to a greater extent [43]. As an interface between motivation and cognition, striatal dopamine has been associated with cognitive flexibility [44]. However, this proposal is only partially supported in the present study, since vigorous-intensity PA was positively associated with fluency and flexibility but not novelty, while the latter was positively associated with walking, and none of these measures were associated with moderate-intensity PA. The neurobiological and psychological mechanism by which walking enhances novelty of divergent thinking is unclear and remains to be investigated. Nevertheless, this finding is consistent with a recent report that a brief walk on a treadmill or outdoors can boost divergent thinking in terms of novelty on the AUT [16]. In this study, subjects generated more novel responses on a 4-min AUT while walking on a treadmill compared to while staying seated. Furthermore, the brief walk also enhanced subsequent divergent thinking such that those walked on the treadmill for the first AUT also performed better on a second AUT while staying seated compared to those sat for both AUTs. These findings may provide a scientific account of the popular “walking meetings” valued by the academic and managerial fields [45,46]. Although with different tasks to evaluate convergent thinking, both the current study (using matchstick puzzles) and Oppezzo and Schwartz (2014) [16] (using the compound remote associates test) failed to find any significant association between PA and convergent thinking. It remains for future studies to confirm this lack of association and clarify the underlying reasons.

The WHO Guidelines on Physical Activity and Sedentary Behavior has recommended vigorous-intensity and moderate-intensity PA as the primary PA for people of all age groups, including children, adolescents, adults, and older adults [1]. However, in the case of creative thinking, together with recent evidence [16], the current study indicates that walking may be more valuable than vigorous- and moderate-intensity PA, since only walking is associated with novelty of divergent thinking. If these findings are replicated by more robust studies, they will have great implications for cognitive and performance enhancement.

Meanwhile, we found that regular PA was not associated with the acute aerobic exercise intervention effects. The interaction effect of regular and acute PA on mood has been reported and debated (e.g., [28,30,32]). Our current findings are consistent with [32] that the acute effects might be independent of the levels of regular PA. Unexpectedly, however, we found that the amount of moderate-intensity PA was positively associated with the matchstick retest measure of convergent thinking after the control intervention in which subjects quietly read mood-neutral materials while staying seated. This result was not an artifact due to a poorer performance at pre-test. These findings suggest the possibility that when solving insight problems, subjects with a greater amount of moderate-intensity PA might be more likely to benefit from a “second thought” after a physically inactive but not physically active “incubation” period [47]. This speculation, however, remains to be tested by future well-designed experimental studies.

Several limitations of the present study have to be noted. Firstly, our subjects were undergraduate students, which prohibits us from generating our findings to other populations. Secondly, our sample size was relatively small, in particular for the analysis on the interaction between regular and acute PA effects. Thirdly, given our small sample size, we did not probe the potential sex difference in the associations between regular PA and creative thinking. Fourthly, we used only a self-report scale (i.e., the IPAQ) to measure PA. Although the validity of the scale has been established [33], there remains the possibility that the self-reported data are biased by social desirability. Future investigations are required to include measures of social desirability bias or confirm our findings with more accurate, objective measures of PA. We hope the current exploratory study may provide useful insights for future studies with larger sample sizes and more divergent populations to advance our understanding of the associations between regular PA and creative thinking.

## 5. Conclusions

In a sample of young adults, we found that regular vigorous-intensity PA was positively associated with fluency and flexibility, while regular walking was positively associated with novelty of divergent thinking. None of the different intensities of regular PA were associated with convergent thinking or the acute aerobic exercise intervention effects on divergent and convergent thinking. These findings suggest that engaging in regular vigorous-intensity PA and walking may be employed as useful strategies to enhance different aspects of divergent thinking in daily life. Whereas the WHO recommends vigorous-intensity and moderate-intensity PA as the primary PA for people of all age groups, the current study indicates that in the case of creative thinking, walking may be more valuable than vigorous- and moderate-intensity PA, since only walking is associated with novelty of divergent thinking. If these findings are replicated by more robust studies, they will have great implications for cognitive and performance enhancement.

## Figures and Tables

**Table 1:** Multiple linear regression results using intensity-specific regular PA to predict creative thinking at baseline.

Dependent Variables		Divergent Thinking (AUT)			Convergent Thinking
		Fluency	Flexibility	Novelty	Matchstick Pre-Test
Independent variables	Vigorous	0.382 *	0.342 *	0.253	0.062
	Moderate	0.031	0.046	0.080	−0.028
	Walking	0.262	0.283	0.378 *	−0.039
	F(3,36)	3.062	2.774	3.013	0.070
Model statistics	R^2^	0.203	0.188	0.201	0.006
	*p*	0.040 *	**0.055 +**	0.043 *	0.976

Note: Standardized coefficients are presented here. The unstandardized coefficients and their 95% confidence intervals are reported in Table S3. Vigorous refers to vigorous-intensity PA; moderate refers to moderate-intensity PA. * *p* < 0.05; + *p* < 0.06.

**Table 2:** Multiple linear regression results using intensity-specific regular PA to predict the intervention effects on creative thinking.

Dependent Variables		Divergent Thinking (AUT)			Convergent Thinking	
		△Fluency	△Flexibility	△Novelty	△CPS	Matchstick Retest
Exercise group						
Independent variables	Vigorous	−0.214	−0.152	−0.191	0.177	0.297
	Moderate	−0.071	−0.106	−0.069	0.3369	0.033
	Walking	−0.078	−0.038	−0.166	0.051	−0.329
	F(3,16)	0.310	0.215	0.330	1.042	1.302
	R^2^	0.055	0.039	0.058	0.163	0.246
	*p*	0.818	0.885	0.804	0.401	0.319
Control group						
Independent variables	Vigorous	−0.157	−0.263	−0.168	−0.228	−0.170
	Moderate	0.301	0.204	0.011	−0.029	0.566 *
	Walking	−0.144	0.207	−0.305	−0.265	−0.421
Model statistics	F(3,16)	0.703	0.789	0.697	0.725	3.554
	R^2^	0.116	0.129	0.116	0.120	0.492
	*p*	0.564	0.517	0.567	0.552	**0.051 +**

Note: Standardized coefficients are presented here. The unstandardized coefficients and their 95% confidence intervals are reported in Table S4. Vigorous refers to vigorous-intensity PA; moderate refers to moderate-intensity PA. For matchstick retest, F(3,12) for exercise, F(3,11) for control. * *p* < 0.05; + *p* < 0.06.

## Data Availability

The data that support the findings of this study are available from the corresponding author upon reasonable request.

## Supplementary Materials

The following are available online at https://www.mdpi.com/article/10.3390/brainsci11081046/s1, Table S1: Summary of previous studies on the effects of regular physical activity on creative thinking: interventional studies; Table S2: Summary of previous studies on the effects of regular physical activity on creative thinking: observational studies; Figure S1: Normal P-P plot of regression standardized residual of multiple linear regressions shown in Table 1; Figure S2: Normal P-P plot of regression standardized residual of multiple linear regressions shown in Table 2; Table S3: The unstandardized coefficients and their 95% confidence intervals for multiple linear regressions shown in Table 1; Table S4: The unstandardized coefficients and their 95% confidence intervals for multiple linear regressions shown in Table 2.
