# Supplementary material for: Regular Vigorous-Intensity Physical Activity and Walking Are Associated with Divergent but not Convergent Thinking in Japanese Young Adults
Source: Brain Sci. 2021 Aug 6;11(8):1046. doi: 10.3390/brainsci11081046 (PMC8392324; doi:10.3390/brainsci11081046)
Supplement: Supplementary file 1 [file brainsci-11-01046-s001.zip › brainsci-1335762-supplementary.pdf]

Supplementary material

Table S1. Summary of previous studies on the effects of regular physical activity on creative thinking: Interventional studies

	Subjects	Design	Exercise intervention	Control intervention	Findings			
					Divergent thinking: AUT	Divergent thinking: other tests	Convergent thinking	Other findings on exercise and/or creativity
Gondola & Tuckman 1985	Students	Quasi-experimental: Ex vs Con at post-test	n=26; 20-min running, twice a week, 8 weeks	n=23; Health education lecture	Fluency: ↑	Remote Consequences: ↑	Matchsticks: ns	-
Gondola 1986	College students	Quasi-experimental: Ex vs Con at post-test	Spring Exercise: n=23; 20-min running, twice a week, 8 weeks<br>Summer Exercise: n=19; 20-min running, twice a week, 6 weeks	Control: n, unreported; health/fitness-oriented class without any physical activity in Spring	Fluency: ↑ for both Exercise groups	Remote Consequences: ↑ for Summer Exercise only	Matchsticks: *ns* between Spring Exercise and Control	Fitness (1.5-mile run): ↑ for both Exercise groups
Tuckman & Hinkle 1986	4-6th grade students (*n*=154)	RCT: Ex vs Con at post-test	30-min running, three times a week, 12 weeks	Regular physical education, including basketball, volleyball, and occasional jogging	Fluency: ↑	-	-	Intervention*Sex interaction on AUT: *ns*<br>Intervention*Grade interaction on AUT: *ns*<br>Fitness (800-m run): ↑
Hinkle et al., 1993	8th grade students (*n*=85)	RCT: Ex vs Con % post-pre change	Running, five times a week, 8 weeks	Regular physical education, including badminton, table	-	TTCT:<br>Figural fluency ↑<br>Figural flexibility ↑<br>Figural originality ↑	-	Intervention*Grade interaction on TTCT: *ns*<br>Fitness (800-m run): ↑<br>Mood and self-esteem

				tennis, volleyball		Figural elaboration *ns*<br>Verbal fluency *ns*<br>Verbal flexibility *ns*<br>Verbal originality *ns*		related measures: *ns*<br>Significant sex difference in figural elaboration, verbal originality: Female > Male
Herman-Tofler & Tuckman 1998	3rd grade students (*n*=52)	RCT: Ex vs Con at post-test	25-min aerobics with energetic music, three times a week, 8 weeks	Regular physical education about various racket sports, including practice	-	TTCT:<br>Figural fluency ↑ (*d*=0.71)<br>Figural originality *ns*<br>Figural elaboration *ns*	-	Fitness (800-m run): *ns*<br>Self-perception of athletic competence, social acceptance, global self-worth, etc.: *ns*
Pedro Ángel et al., 2021	3rd-6th grade students (*n*=140)	RCT: Ex vs Con group*time interaction	30-min active recess program, three times a week, 10 weeks	No intervention	-	PIC-N:<br>Narrative fluency ↑<br>Narrative flexibility ↑<br>Narrative originality ↑<br>Graphic design ↑<br>Graphic shadow and color ↑<br>Graphic details *ns*	-	School aptitudes: ↑<br>Cognitive flexibility (TMT-B): ↑<br><br>Correlation between improvement in cognitive flexibility and improvement in VO_2max (*r*=-0.29*)

AUT: Alternate Uses Test; **TTCT**: Torrance Tests of Creative Thinking; **PIC-N**: Prueba de Imaginación Creativa – Niños ***ns***: nonsignificant.
Literature search strategy: *database*: Pubmed; *search field*: Title/Abstract; *search terms*: (exercise OR physical activity OR walking OR running OR cycling OR aerobic training OR fitness training OR resistance training) AND (regular OR habit OR program OR chronic OR days OR week OR month) AND (creativity OR divergent thinking OR convergent thinking OR Alternate Uses OR Guilford OR Torrance OR remote associates test OR cognitive flexibility OR analogy OR insight problem solving OR metaphors); *last search and confirmation date*: 2021/07; a cross-reference search was also conducted. *d*: Cohen's d. *r*: correlation coefficient. *$p<0.05$. In addition to the studies listed here, we also found another interventional study that evaluated creative thinking in terms of creating analogies using the Similarities subtest of the Wechsler Adult Intelligence Scale-Revised (Kamegaya, T., Araki, Y., Kigure, H., Long-Term-Care Prevention Team of Maebashi City, & Yamaguchi, H. (2014). Twelve-week physical and leisure activity programme improved cognitive function in community-dwelling elderly subjects: a randomized controlled trial. Psychogeriatrics, 14(1), 47-54.).

Table S2. Summary of previous studies on the effects of regular physical activity on creative thinking: Observational studies

	Subjects	Design	Regular physical activity measures	Creative thinking measures	Findings: association between regular physical activity and creative thinking	Other findings on physical activity and/or creativity
Cavallera et al. 2011	University students (*n*=61)	Cross-sectional	Self-reported number of hours of sport activity per week	Divergent thinking: TTCT figural series	Elaboration: correlation coefficient *r*=0.31*<br>Fluency: *ns*<br>Flexibility: *ns*<br>Originality: *ns*	Morningness-eveningness personality did not affect the association
Rominger et al., 2020	University students (*n*=79)	Cross-sectional	ActiGraph®, over 5 consecutive days	Divergent thinking: Combined measure of AUT originality and TTCT figural originality	Total everyday bodily movement: correlation coefficient *r*=0.32**<br>No to light activity: *r*=-0.29*<br>Moderate activity: *r*=0.26*<br>Vigorous activity: *r*=0.15, *ns*<br>Very vigorous activity: *r*=0.20, *ns*	Positive-activated affect did not mediate the association between everyday bodily movement and creativity
Nakagawa et al., 2020	Young adults (*n*=49)	Cross-sectional	IPAQ-short: days of walking, moderate, and vigorous intensity physical activity	Convergent thinking: Insight problem solving (matchsticks, nine-dot puzzle, and coin puzzle)	*ns* (regression model including all three regular exercise measures as independent variables)	Moderate intensity physical activity predicted working memory and coping strategies<br>Vigorous intensity physical activity predicted coping strategies and psychological wellbeing
Piya-amornphan et al., 2020	Students of age 6-17 (*n*=1447)	Cross-sectional	TPACS-SQ: Number of days with at least 60 min of active play in the past week	Divergent thinking: TCT-DP	Age 6-9 years: r=0.010, *ns*<br>Age 10-13 years: r=0.014, *ns*<br>Age 14-17 years: r=0.148**	No correlation between creativity and time spent with family and peers for all age groups<br>No correlation between creativity and time in sedentary behavior for all age groups

AUT: Alternate Uses Test; **TTCT**: Torrance Tests of Creative Thinking; **TCT-DP**: Test for Creative Thinking-Drawing Production; **TPACS-SQ**: Thailand Physical Activity Children Survey-the Student Questionnaire; *r*: correlation coefficient. ***ns***: nonsignificant. *p<0.05, **p<0.01. We also identified two additional cross-sectional study (Rominger, C., Papousek, I., Fink, A., Perchtold, C. M., Lackner, H. K., Weiss, E. M., & Schwerdtfeger, A. R. (2019). Creative challenge: Regular exercising moderates the

association between task-related heart rate variability changes and individual differences in originality. Plos one, 14(7), e0220205; Jung, M., Kim, H. S., Loprinzi, P. D., & Kang, M. (2021). Serial-multiple mediation of enjoyment and intention on the relationship between creativity and physical activity. AIMS neuroscience, 8(1), 161.). Rominger et al., 2019 tested a regression model including task-related HRV change, the amount of regular exercise, and their interaction as independent variables to predict AUT originality and found that the amount of regular exercise moderated the association between task-related HRV change and originality. Jung et al., 2021 employed the Kaufman Domains of Creativity Scale (K-DOCS) measuring creative behavior in the domains of self/everyday creativity, scholarly creativity, performance creativity, mechanical creativity, and artistic creativity. This study reported a positive correlation between the amount of PA per week and total creativity ($r=0.60$, $p<0.01$).

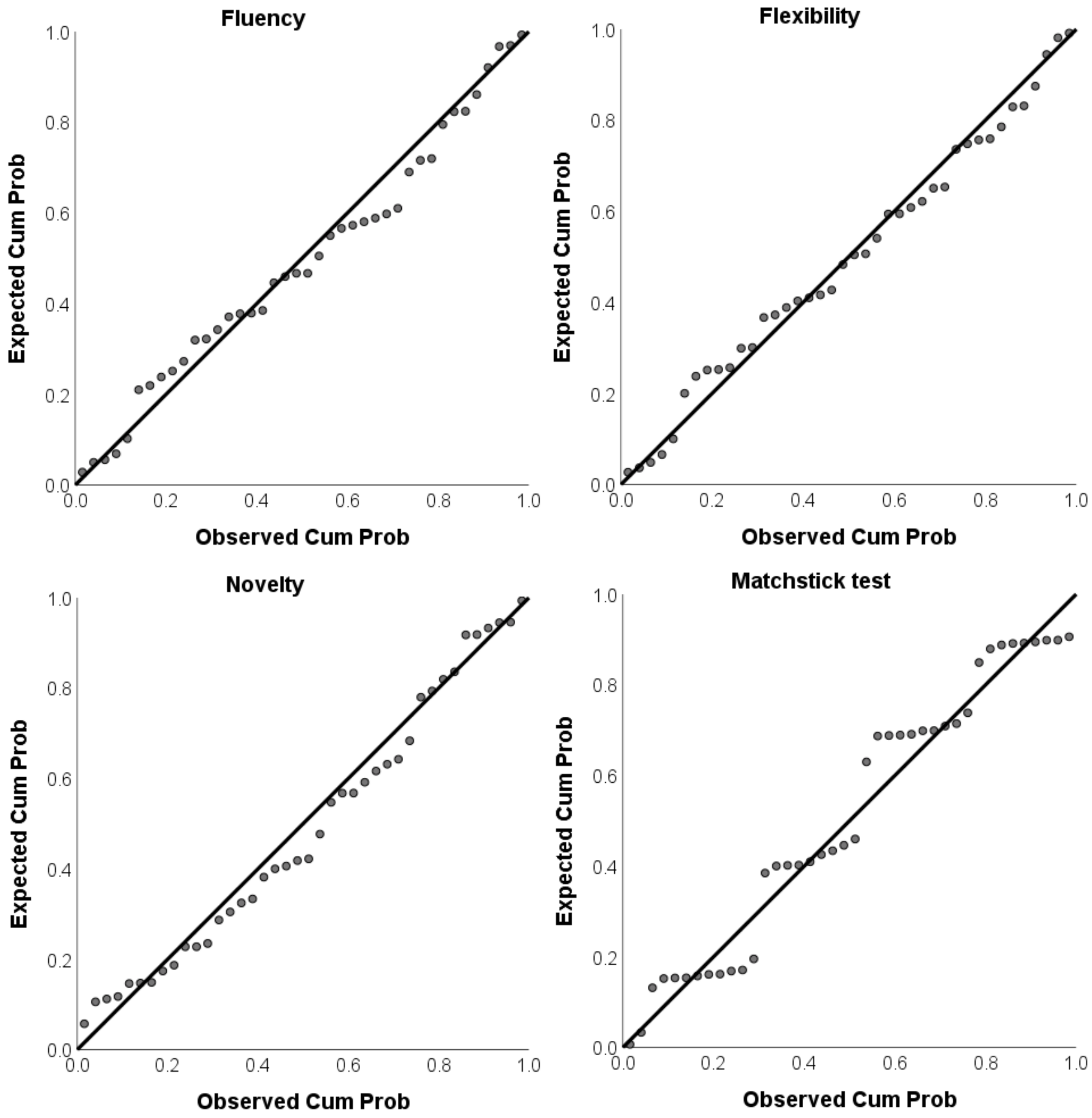

Figure S1. Normal P-P plot of regression standardized residual of multiple linear regressions shown in Table 1.

Exercise

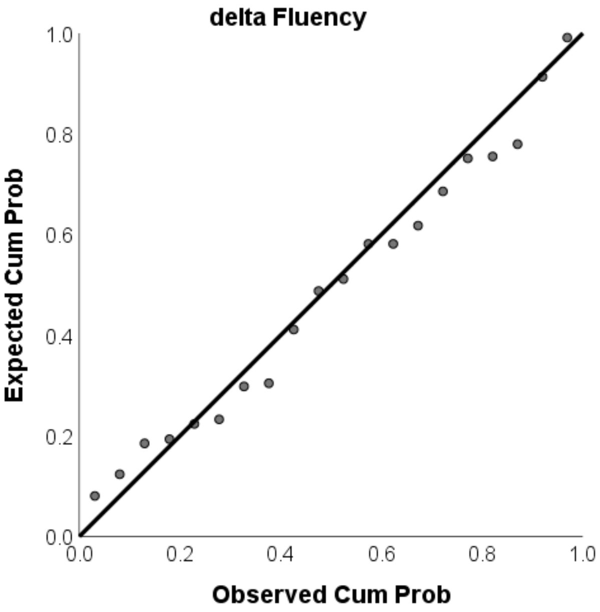
delta Fluency
Expected Cum Prob
Observed Cum Prob
0.0
0.2
0.4
0.6
0.8
1.0

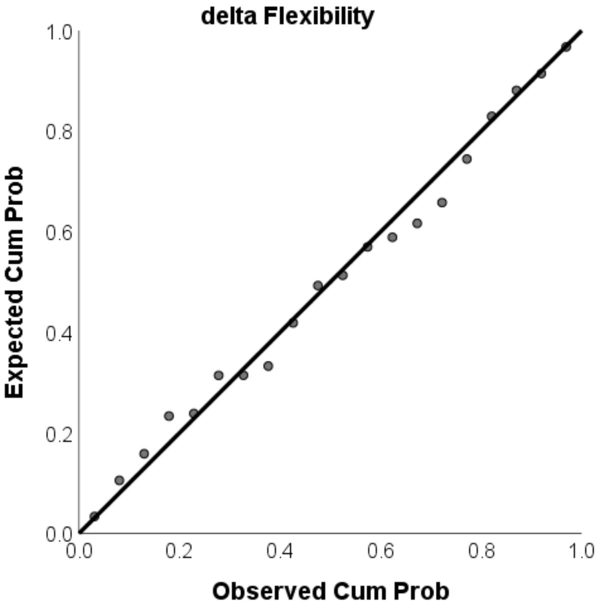
delta Flexibility
Expected Cum Prob
Observed Cum Prob
0.0
0.2
0.4
0.6
0.8
1.0

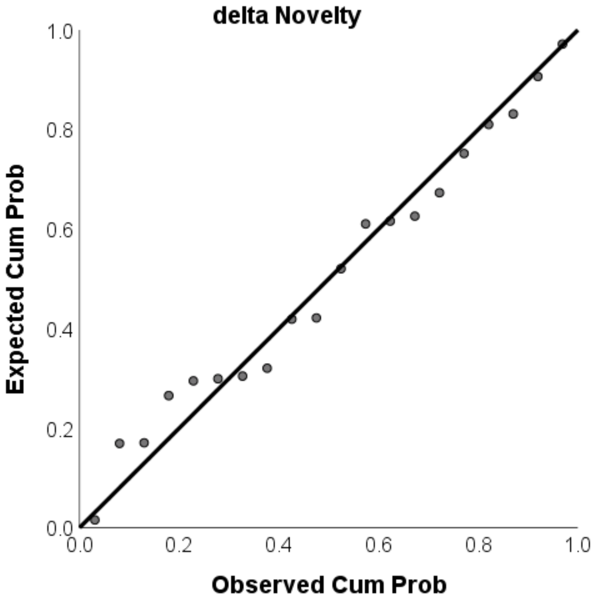
delta Novelty
Expected Cum Prob
Observed Cum Prob
0.0
0.2
0.4
0.6
0.8
1.0

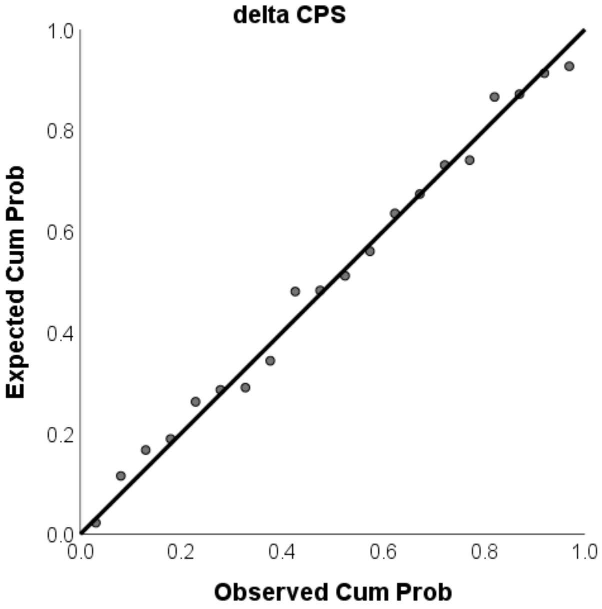
delta CPS
Expected Cum Prob
Observed Cum Prob
0.0
0.2
0.4
0.6
0.8
1.0

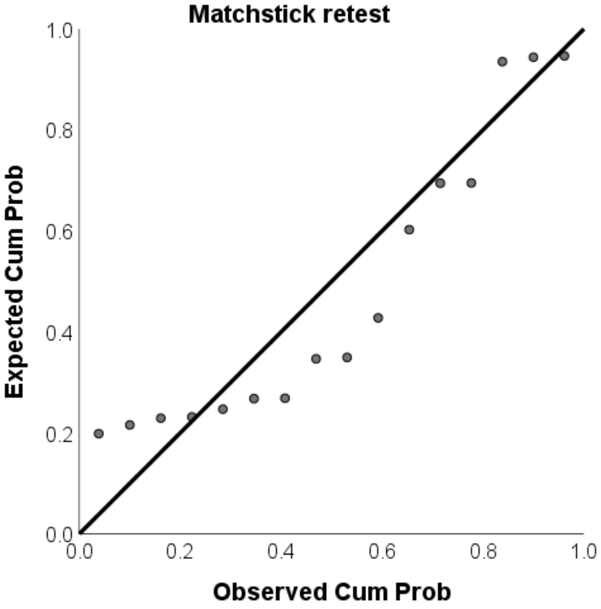
Matchstick retest
Expected Cum Prob
Observed Cum Prob
0.0
0.2
0.4
0.6
0.8
1.0

Control

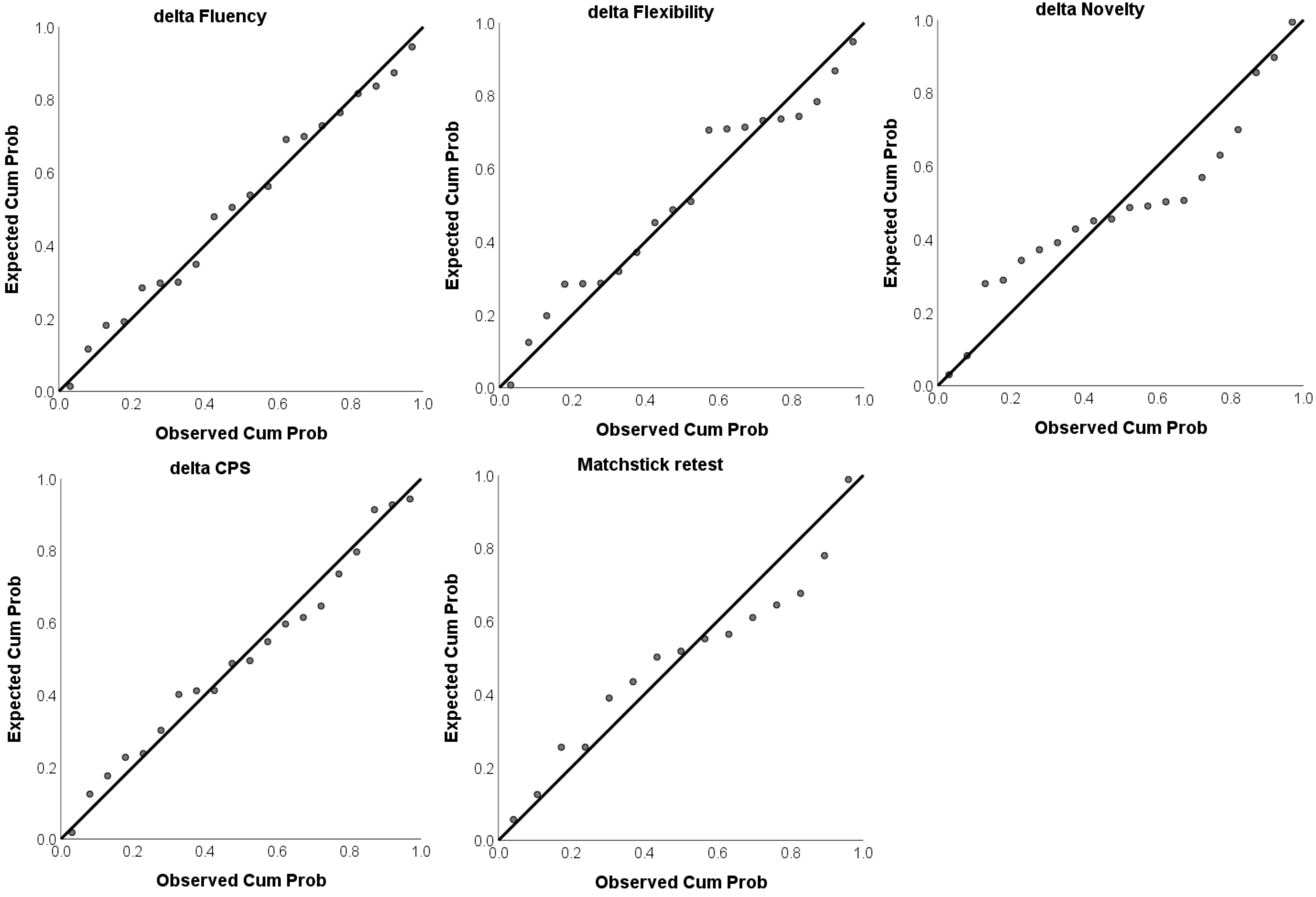

Figure S2. Normal P-P plot of regression standardized residual of multiple linear regressions shown in Table 2.

Table S3. The unstandardized coefficients and their 95% Confidence Intervals for multiple linear regressions shown in Table 1.

**Dependent variables**		**Divergent thinking (AUT)**			**Convergent thinking**
		**Fluency**	**Flexibility**	**Novelty**	**Matchstick pre-test**
Independent variables	Vigorous	**0.007(0.001, 0.012)**	**0.006(0.001, 0.011)**	0.002(0.000, 0.005)	0.00006(0.000, 0.000)
	Moderate	0.000(-0.004, 0.004)	0.001(-0.003, 0.004)	0.001(-0.002, 0.003)	-0.00002(0.000,0.000)
	Walking	0.005(-0.001,0.011)	0.005(0.000, 0.011)	**0.004(0.001, 0.007)**	-0.00004(0.000,0.000)

Table S4. The unstandardized coefficients and their 95% Confidence Intervals for multiple linear regressions shown in Table 2.

**Dependent variables**		**Divergent thinking (AUT)**			**Convergent thinking**	
		**△Fluency**	**△Flexibility**	**△Novelty**	**△CPS**	**Matchstick retest**
Exercise group						
Independent variables	Vigorous	-0.005(-0.018, 0.008)	-0.003(-0.014, 0.008)	-0.002(-0.008, 0.004)	0.000(-0.001, 0.001)	0.001(-0.001, 0.002)
	Moderate	-0.001(-0.008, 0.006)	-0.001(-0.007, 0.005)	0.000(-0.004, 0.003)	0.000(0.000, 0.001)	0.00005(-0.001,0.001)
	Walking	-0.002(-0.013, 0.010)	-0.001(-0.010, 0.009)	-0.002(-0.007, 0.004)	-0.00007(-0.001,0.001)	-0.001(-0.002, 0.001)
Control group						
Independent variables	Vigorous	-0.002(-0.008, 0.004)	-0.003(-0.008, 0.003)	-0.002(-0.006, 0.003)	0.000(-0.001, 0.000)	0.000(-0.001, 0.001)
	Moderate	0.003(-0.002, 0.008)	0.002(-0.003, 0.007)	-0.00009(-0.004, 0.007)	-0.00004(-0.001, 0.001)	**0.001(0.000,0.001)**
	Walking	-0.002(-0.009, 0.005)	-0.003(-0.009, 0.004)	-0.003(-0.009, 0.002)	0.000(-0.001, 0.000)	-0.001(-0.001,0.000)
